# Cytokine Network in Adults with Falciparum Malaria and HIV-1: Increased IL-8 and IP-10 Levels Are Associated with Disease Severity

**DOI:** 10.1371/journal.pone.0114480

**Published:** 2014-12-11

**Authors:** Aase Berg, Sam Patel, Miguel Gonca, Catarina David, Kari Otterdal, Thor Ueland, Ingvild Dalen, Jan T. Kvaløy, Tom E. Mollnes, Pål Aukrust, Nina Langeland

**Affiliations:** 1 Department of Medicine, Stavanger University Hospital, Stavanger, Norway; 2 Department of Medicine, The Central Hospital of Maputo, Maputo, Mozambique; 3 Department of Clinical Science, University of Bergen, Bergen, Norway; 4 Research Institute of Internal Medicine, Oslo University Hospital Rikshospitalet, Oslo, Norway; 5 Research Department, Stavanger University Hospital, Stavanger, Norway; 6 Department of Mathematics and Natural Sciences, University of Stavanger, Stavanger, Norway; 7 Section of Clinical Immunology and Infectious Diseases, Oslo University Hospital Rikshospitalet, Oslo, Norway; 8 Faculty of Medicine, University of Oslo, Oslo, Norway; 9 K.G. Jebsen Inflammatory Research Center, University of Oslo, Oslo, Norway; 10 Institute of Immunology, Oslo University Hospital Rikshospitalet, Oslo, Norway; 11 Research Laboratory Nordland Hospital, Bodø, Norway; 12 Faculty of Health Sciences, University of Tromsø, Tromsø, Norway; 13 Department of Medicine, Haukeland University Hospital, Bergen, Norway; Liverpool School of Tropical Medicine, United Kingdom

## Abstract

**Background:**

Co-infection with malaria and HIV increases the severity and mortality of both diseases, but the cytokine responses related to this co-infection are only partially characterised. The aim of this study was to explore cytokine responses in relation to severity and mortality in malaria patients with and without HIV co-infection.

**Methods:**

This was a prospective cross-sectional study. Clinical data and blood samples were collected from adults in Mozambique. Plasma was analysed for 21 classical pro- and anti-inflammatory cytokines, including interleukins, interferons, and chemokines.

**Results:**

We included 212 in-patients with fever and/or suspected malaria and 56 healthy controls. Falciparum malaria was diagnosed in 131 patients, of whom 70 were co-infected with HIV-1. The malaria patients had marked increases in their cytokine responses compared with the healthy controls. Some of these changes, particularly interleukin 8 (IL-8) and interferon-γ-inducing protein 10 (IP-10) were strongly associated with falciparum malaria and disease severity. Both these chemokines were markedly increased in patients with falciparum malaria as compared with healthy controls, and raised levels of IL-8 and IP-10 were associated with increased disease severity, even after adjusting for relevant confounders. For IL-8, particularly high levels were found in malaria patients that were co-infected with HIV and in those who died during hospitalization.

**Interpretations:**

Our findings underscore the complex role of inflammation during infection with *P. falciparum,* and suggest a potential pathogenic role for IL-8 and IP-10. However, the correlations do not necessarily mean any causal relationship, and further both clinical and mechanistic research is necessary to elucidate the role of cytokines in pathogenesis and protection during falciparum malaria.

## Introduction

*Human Immunodeficiency Virus* (HIV) 1 and *Plasmodium falciparum* malaria are two of the major lethal infectious diseases in sub-Saharan Africa, with 1.47 million and 1.13 million deaths, respectively, in 2010 [1], [2]. Co-infection with malaria and HIV presents challenges in both diagnosis and therapy and results in increased severity and mortality of both infections [3]–[5].

Infection with *P.falciparum* is often associated with the activation of several inflammatory pathways, resulting in systemic and local inflammatory responses that involve the production of a wide range of cytokines,- from several cell types including leukocyte subsets, endothelial cells and various tissues cells such as macrophages. This inflammatory response during malaria infection could activate anti-microbial responses in macrophages, T cells, granulocytes and endothelial cells and thereby be beneficial for the host [6]. On the other hand, an overwhelming inflammatory response could be detrimental for the host by contributing to tissue destruction and excessive systemic inflammation that could contribute to morbidity and mortality during falciparum malaria. Rodent studies indicate that the malaria parasites themselves produce their own cytokines, which interfere with the host's immune response [7], but characterisation of the different actors in the activated cytokine network during clinical falciparum malaria is not fulfilled.

Although the immunological hallmark of HIV infection is a loss of CD4^+^ T cells and the development of severe immunodeficiency, it is also characterised by a state of chronic inflammation [8], and this non-resolving inflammation will further contribute to immunodeficiency through mechanisms such as immune exhaustion [9], [10]. This persistent low-grade immune activation will affect immune responses to co-infection with other microbes, including *P. falciparum.* However, in what way co-infection with HIV influences cytokine responses during falciparum malaria is still only partly known. In addition, although there are numerous studies on cytokine response during infection with *P. falciparum*, less is known about the more detailed cytokine network and the relative importance of different mediators with regard to disease severity and mortality in malaria patients.

Mozambique has one of the highest global incidences of co-infection with HIV-1 and falciparum malaria, with a population HIV-1 prevalence of 11.5% that is 22.5% in Maputo (adults 15–49 years, 2009) and a malaria parasite prevalence of 27.7% in small children in Southern Mozambique (2007) [11], [12]. To characterise inflammatory responses during infection with *P. falciparum* with and without co-infection with HIV-1, we analysed a wide range of cytokines and related inflammatory mediators in blood samples obtained at admission from adult patients admitted to Central Hospital of Maputo in Mozambique with fever and/or suspected malaria. The levels of the different mediators were related to disease severity, mortality, and co-infection with HIV.

## Methods

### Study area and participants

The Central Hospital of Maputo is a public teaching hospital for Maputo's 1.2 million citizens and a national referral hospital for Mozambique's population of 22 million. From 8 January 2011 to 31 March 2011 and from 7 November 2011 to 14 March 2012, we conducted a prospective cohort study in all patients consecutively admitted to the Medical Emergency Department in the Central Hospital in Maputo every week from Sunday to Friday, as previously described [13]. Most of the patients and most of the healthy controls came from the more peripheral suburbs of Maputo city, including marshland. Malaria is meso-endemic and most frequent from November to April, with *P. falciparum* accounting for 95-100% of the malaria cases [12], [13]. The study was designed and performed according to the Helsinki Declaration, as adopted by the 59th WMA General Assembly, Seoul, Republic of Korea, October 2008, and was approved by The National Ethical Committee at the Ministry of Health in Mozambique and the Regional Ethical Committee in Eastern Norway. A signed consent or fingerprint was obtained from each patient or next of kin and from the healthy controls. Management of the patients was carried out according to the local standard of care by the hospital's doctors and staff.

### Inclusion criteria

All non-pregnant adults ≥18 years with an axillary temperature ≥38.0°C and/or suspected malaria were included, provided written consent. As controls, we included health care workers at the hospital, friends, and family members who reported a subjective feeling of wellbeing and had a healthy appearance, as evaluated by the researchers. Female healthy controls were not included if suspected or confirmed pregnant. We defined “Suspected malaria” as a history of fever, chills, headache, mental confusion, vomiting and/or diarrhoea, dyspnea, myalgia, and/or general malaise, provided that there were no other symptoms, findings on clinical examination, or additional diagnostic tests indicating other infections. Additional diagnostic tests and exams were basic laboratory tests (e.g., Hemoglobin (Hb), WBC, differential count, ESR, AST, ALT, ALP, bilirubin, L DH, creatinine); other blood tests (e.g., bacteriological and fungal culture and antigen/antibodies for HIV-1, HIV-2, CMV, EBV, hepatitis B and C); urine analysis (stix, micro, culture); Cerebrospinal fluid (CSF) analysis (erythrocytes, WBC, differential count, protein, glucose, chloride, syphilis and cryptococcal tests, bacteriological and fungal culture); sputum analysis for M. Tuberculosis with microscopy (AAFB) and culture; stool analysis (e.g., microscopy for ova and cysts, bacteriological culture); and cytological/histological and different radiological exams, if indicated. “Malaria positive” (confirmed malaria) was defined as a patient with a positive malaria PCR test. One patient who died with a positive malaria PCR test was categorised as a non-malarial death, because he had a history and clinical findings more compatible with tuberculous meningoencephalitis as the cause of death. No malaria PCR was performed for two patients: they had a positive HRP2-antigen test and were slide-positive with parasitemia 3+ and 5+, respectively, and were defined as malaria positive. HIV-infected patients were defined by a positive HIV serological test and/or a positive HIV PCR test. Malaria severity was categorised according to the number of criteria fulfilled for severe malaria, as defined by the WHO and adjusted to what could be measured locally, as previously reported [13], [14]. The HIV disease severity was categorised according to the WHO HIV clinical staging, I–IV [15].

### Clinical data and blood sampling

We recorded a predefined set of clinical data from the patients' files, which was performed as part of the routine clinical examination on admission and on follow-up during the hospital stay. The details are published elsewhere [13]. Blood samples from the patients and controls were collected from a pre-alcohol-cleaned peripheral vein and drawn into pyrogenic-free tubes with EDTA as a anticoagulant (plasma) or with no additive (serum).

The EDTA tubes were immediately put on melting ice and centrifuged within 30 minutes at 2000x*g* for 20 minutes to obtain platelet-poor plasma. The serum tubes were allowed to clot at room temperature prior to being centrifuged at 1000x*g* for 10 minutes. The serum and plasma samples were stored in multiple aliquots at −20°C for 24 hours before being placed at −80°C. The samples were thawed only once.

### Laboratory analyses

According to the hospital routine and in line with standard procedures in the laboratory of the Central Hospital of Maputo HIV-test (Determine, Abbot Japan Co., Ltd. and Unigold, Trinity Biotech plc, Bray, Ireland), HRP-2 Rapid Diagnostic Test for malaria (RDT) (2010–2011 First Response Malaria antigen *P. falciparum*, Premium Medical Corporation Ltd., Daman, India; 2011–2012 ICT Malaria P.f., ICT Diagnostics Cape Town, South Africa), malaria thick blood slides (Giemsa 20% for 5 minutes), and other routine laboratory tests were performed. Parasitemia was categorised in a thick smear as 1+ when 1–10 parasites/100 fields, 2+ when 11–100 parasites/100 fields, 3+ when 1–10 parasites/field, 4+ when 11–100/field, and 5+ when >100 parasites/field [16]. In addition, blood samples were taken separately for malaria and HIV PCR analyses, as previously described [13]. An HCG-urine pregnancy test was performed for the female patients of fertile age (Quick Vue, Quidel Corp., San Diego, CA, USA).

### Cytokine assays

We analysed the plasma levels of interleukins (IL)-1β, IL-1 receptor antagonist (IL1-ra), IL-2, IL-4, IL-5, IL-6, IL-7, IL-9, IL-10, IL-12 (p70), IL-13, IL-15, IL-17, interferon (IFN)-γ and tumour necrosis factor (TNF) as well as chemokines IL-8/CXCL 8, eotaxin 1/CCL11, IFN-γ-inducing protein 10 (IP-10/CXCL10), monocyte chemotactic protein-1 (MCP-1/CCL2), and macrophage inflammatory proteins 1α (MIP-1α/CCL3) and MIP-1β/CCL4 by a multiplex cytokine assay (Bio-Plex Human Cytokine 27-Plex Panel; Bio-Rad Laboratories Inc., Hercules, CA, USA) according to the instructions from the manufacturer. Cytokines that were not detectable (all or most values below the lower detection limit) were excluded from further analysis (i.e., IL-1β, IL-2, IL-4, IL-5, IL-12, IL-13, and IL-15). There were no cytokine measurements above the upper detection limit.

### Statistical analysis

For the cytokine data, we report the means, medians, and 25^th^ and 75^th^ percentiles. Differences in categorical variables such as sex and HIV-status were tested by chi-square tests. Differences in cytokine distributions between the groups of study participants were tested by Mann-Whitney tests. Spearman rank correlation was used for calculating correlations between cytokines in all malaria patients (S1 Figure). Because this was an exploratory study with many inter-dependent markers, we used no statistical corrections for multiple comparisons. For multivariate analyses, due to high correlations among many of the cytokines, ordinary multivariable logistic regression was not suitable. Instead we used Pelora, a method constructed for supervised clustering in situations with strong correlations among predictors (here i.e. cytokines), and many predictors compared to the number of individuals (Pelora), which is recommended for such cytokine analyses [17], [18], i.e. exactly the challenges we have in cytokine data [17]. Pelora finds groups of cytokines characterizing the property we are searching for, as which group of cytokines are characteristic for the difference between all malaria patients compared to healthy controls, for HIV positive malaria patients compared to the HIV negative malaria patients and for those with severe malaria compared to the patients with uncomplicated malaria. The ability of a cytokine group to distinguish between patient groups, or patients versus controls, is given as the “predictive ability”, measured by the area under the operator characteristic (ROC) curve [19], and where the closer to 1,- the better. Each cytokine in a group may have a positive or a negative effect on the patient group membership probability [17], [18]. According to the recommendations for this method, we included clusters into the models until there was a levelling off in the increase in predictive ability, and we adjusted for the effect of age, sex and where relevant HIV status. Prior to Pelora, the cytokine values were log transformed to normalize their skewed distribution, and the 1–2 most extreme values of the most skewed cytokines were adjusted down to 3SD from the mean.

For analyses involving variables for which there were missing observations for certain patients, these subjects were excluded; the number of included patients is indicated in the tables. In the calculation of malaria severity, missing observations were reckoned as “normal” in cases in which the investigation in question, e.g., bilirubin, was performed only on the clinical suspicion of specific organ involvement. Most of the statistical analyses were performed with SPSS-21 (IBM Corporation 1, New Orchard Road, Armonk, New York 10504–1722, USA, 914–499–1900), but the Pelora analysis and the correlations plots were performed in R version 3.0. (http://www.r-project.org) using the R-packages “supclust“and “corrplot”, respectively. There is a validation of all statistical methods used in S1 File.

## Results

### Characteristics of study participants

**Figure 1:**
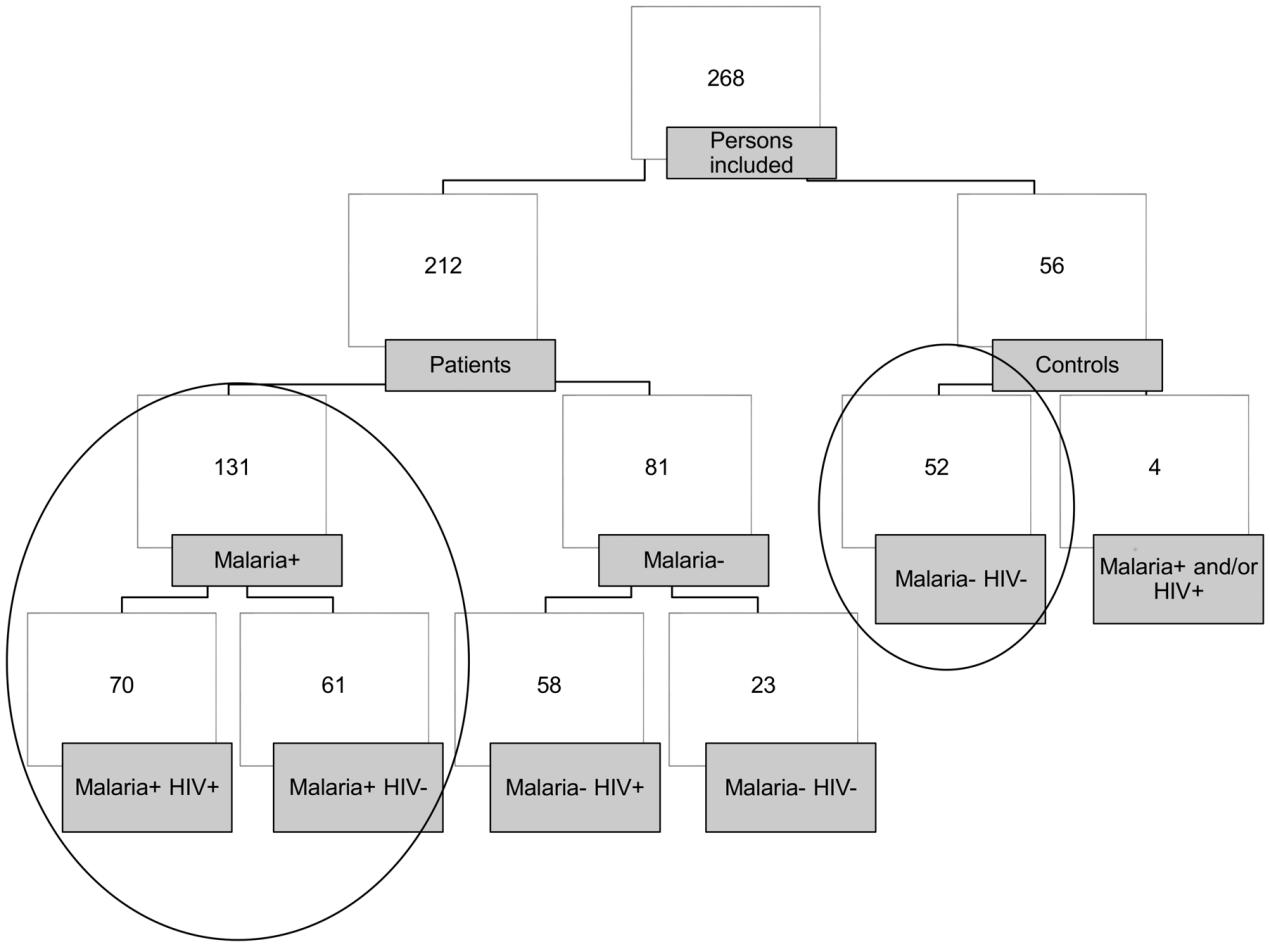
Study population, flow diagram. The focused sub-populations are encircled. Description of study population [13].

A total of 212 non-pregnant adults with fever and/or suspected malaria and 56 healthy controls were included. Of the 212 patients, 131 (62%) had malaria, and 70 of these (53%) were co-infected with HIV-1 (Fig. 1). All malaria patients had *P. falciparum*; two also had double infection, one with *P. vivax* and the other with *P. malariae*. Four of the healthy controls had HIV infection, including one with *P.ovale* in addition. Those were excluded from further analysis rendering 52 healthy controls.

Among the malaria patients and healthy controls, the median ages were 38 years (range 18–79 years) and 26 years (range 18–56 years), respectively: 45% and 41% were women, and 98.5% and 96% were ethnic Mozambicans. There was a significant difference between the malaria patients and healthy controls in relation to age (p = 0.003), but not in relation to sex (p = 0.567). The malaria patients with and without HIV co-infection showed no significant differences in age, sex, and duration of symptoms; 66% of the patients (85/129) had severe malaria defined as one or more severity criteria (missing observations for two patients). The malaria patients with HIV co-infection in general had more severe disease, including in-hospital mortality, as recently reported [13]. Of the malaria patients with HIV co-infection, 59% (41/70) had severe HIV infection, with HIV WHO stage 3 or 4. The HIV-infected patients had a median HIV viral load at 1.8 x 10^4^ (range 0–8.3 x 10^6^) HIV RNA copies/mL plasma (n = 62, missing blood sample analysis for eight patients). Unfortunately, data on the CD4 T-cell counts were lacking in all except 11 patients who had a median of 206 CD4^+^ T cells/µL (range 14–632).

### Overall cytokine response

The cytokine responses were extremely variable both inter- and intra-individually, with levels ranging from below the lower detection limit to a 1000-fold increase above this limit. Within the malaria patient group, several of the cytokines were strongly inter-correlated, irrespective of co-infection with HIV, illustrating the marked changes in and close connections between cytokines during malaria infection (S1 Figure).

### Cytokine levels in malaria patients compared with healthy controls

In general, the malaria patients had a pronounced and broad cytokine response compared with the healthy controls (Table 1). We observed significantly higher levels of IL-1ra, IL-6, IL-8, IL-9, IL-10, eotaxin, IP-10, MCP-1, and MIP-1β. Pelora first indicated an increased IP-10 response as the most important cytokine for differentiating between malaria patients and healthy controls, with a predictive ability of 0.973. The second most important group of cytokines were increased IL-8, IL-10, MIP-1β, and eotaxin and reduced IL-17 and MIP-1α with a high combined estimated predictive ability of 0.969.

**Table 1:** Circulating levels of inflammatory mediators in adult falciparum malaria patients compared with healthy controls.

	Healthy controls (n = 52)			Malaria patients (n = 129)1)				
Mediator	Median	Mean	Q1, Q3	Median	Mean	Q1, Q3	p2)	Pelora3)
IL-1ra	118	171	61, 217	282	1089	145, 676	**<0.001**	
IL-6	8	13	5, 17	33	349	16, 85	**<0.001**	
IL-7	9	14	5, 20	12	18	5, 24	0.476	
IL-8	12	20	6, 26	28	83	15, 54	**<0.001**	X
IL-9	8	11	3, 17	18	24	10, 30	**<0.001**	
IL-10	4	10	2, 13	46	261	16, 135	**<0.001**	X
IL-17	9	31	2, 51	1,68	23	2, 21	0.187	X↓
Eotaxin	6	15	2, 21	28	78	6, 75	**<0.001**	X
INF-γ	80	117	49, 138	92	177	57, 174	0.308	
IP-10	258	428	212, 334	5877	12007	3062, 15230	**<0.001**	X
MCP-1	9	12	6, 16	41	168	18, 152	**<0.001**	
MIP-1α	5	10	3, 13	7	10	4, 11	0.347	X↓
MIP-1β	29	35	21, 44	125	290	69, 276	**<0.001**	X
TNF	42	60	22, 79	43	100	24, 78	0.951	

Boldface type indicates statistical significance. The concentration of the various cytokines is given in pg/mL.

1) Data for two patients were missing.

2) Univariable analysis with the Mann-Whitney test.

3) Calculated by penalised logistic regression, where IP-10 appear as a much stronger indicator of malaria infection compared to the healthy controls, than the other mentioned cytokines. The arrow indicates reduced cytokine levels in malaria patients compared with healthy controls.

### Cytokine levels in malaria patients with or without HIV co-infection

In the malaria patients with HIV co-infection compared with those without, there was a significant increase in the levels of IL-8, eotaxin, and MIP-1α. (Table 2). Pelora extracted high IL-8 and eotaxin and low TNF, with a predictive ability of 0.697. When analysing HIV-infected malaria patients with (n = 13) and without (n = 33) antiretroviral therapy (ART) we saw no significant effects on cytokine levels (data not shown). However, most of the patients on ART had less than three months treatment and the data must therefore be interpreted with caution.

**Table 2:** Circulating levels of inflammatory mediators in adult falciparum malaria patients with and without HIV-1 co-infection.

	Falciparum malaria							
	HIV- (n = 61)			HIV+ (n = 68)3)			p1)	Pelora2)
Mediator	Median	Mean	Q1, Q3	Median	Mean	Q1, Q3		
IL-1ra	273	1354	144, 459	380	861	143, 895	0.243	
IL-6	28	512	15, 81	42	204	19, 103	0.166	
IL-7	12	17	6, 22	13	19	4, 26	0.932	
IL-8	21	77	13, 37	35	89	19, 76	**0.002**	X
IL-9	15	24	9, 29	19	25	10, 33	0.371	
IL-10	40	322	14, 104	63	209	19, 266	0.281	
IL-17	2	17	2, 16	5	28	2, 34	0.100	
Eotaxin	19	95	6, 55	37	62	14, 89	**0.012**	X
INF-γ	92	217	60, 170	90	141	49, 175	0.991	
IP-10	5174	9307	2345, 12371	6637	14573	3603, 19947	0.050	
MCP-1	35	186	16, 122	55	153	21, 177	0.151	
MIP-1α	6	10	4, 8	8	9	5, 14	**0.033**	
MIP-1β	119	322	60, 194	131	264	79, 369	0.183	
TNF	45	141	25, 84	38	63	22, 74	0.666	X↓

Boldface type indicates statistical significance. The concentration of the various cytokines is given in pg/mL.

1) Univariable analysis with the Mann-Whitney test.

2) Calculated by penalised logistic regression. The arrow indicates reduced cytokine levels in HIV co-infected malaria patients compared with patients without HIV.

3) Missing data for two patients.

### Cytokine levels related to disease severity

For patients with severe malaria (n = 84), as defined according to the adjusted WHO criteria [13], [14], there were significantly increased levels of IL-8, IP-10 and MIP-1β compared with the patients with uncomplicated malaria (n = 45) (Table 3). Severe compared to uncomplicated malaria was associated with significantly increased mortality rate with p = 0.017, with missing data for three patients. The Pelora analysis extracted increased IL-8 and IP-10 and reduced MCP-1 levels, with a predictive ability of 0.756 (Table 3).

**Table 3:** Circulating levels of inflammatory mediators in adult patients with uncomplicated and severe falciparum malaria.

	Uncomplicated malaria (n = 45)1)			Severe malaria2) (n = 84)1)				
Mediator	Median	Mean	Q1, Q3	Median	Mean	Q1, Q3	p3)	Pelora4)
IL-1ra	276	1010	128, 485	287	1131	114, 774	0.327	
IL-6	25.0	101	13, 60	40.0	481	18, 92	0.119	
IL-7	12.0	15.7	6, 22	13.0	19.4	4, 26	0.937	
IL-8	23.0	33.0	13, 22	34.0	110	19, 74	**0.002**	X
IL-9	14.0	17.0	7, 26	19.0	28.5	10, 33	0.079	
IL-10	34.0	104	12, 118	53.0	344	20, 181	0.142	
IL-17	1.68	18.02	2, 21	1.68	25.3	2, 23	0.457	
Eotaxin	23.0	106	6, 51	37.0	63.3	7, 82	0.087	
INF-γ	87.0	236	60, 154	98.0	145	54, 178	0.786	
IP-10	4343	6327	1926, 9007	8202	15050	4033, 18920	**0.001**	X
MCP-1	33.0	128	14, 100	53.0	189	21, 160	0.180	X↓
MIP-1α	6.00	7.17	3, 9	7.00	11.1	5, 14	0.096	
MIP-1β	107	173	51, 207	130	352	86, 347	**0.041**	
TNF	44.0	156	27, 72	42.0	70.4	23, 84	0.837	

Boldface type indicates statistical significance. The concentration of the various cytokines is given in pg/mL.

1) Missing data for one patient.

2) Malaria severity criteria adjusted from WHO [15].

3) Univariable analysis with the Mann-Whitney test.

4) Calculated by penalised logistic regression. The arrow indicates reduced cytokine levels in the patients with severe malaria compared with those with uncomplicated malaria.

**Figure 2:**
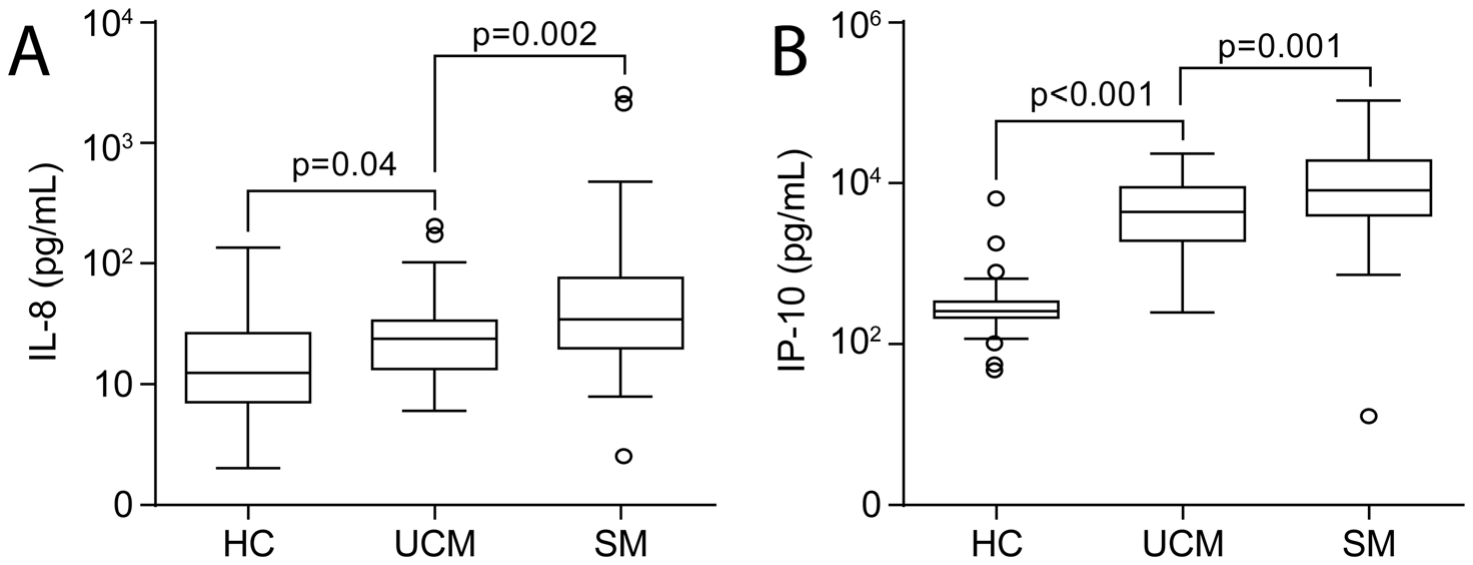
Circulating levels of IL-8 and IP-10 in adult falciparum malaria patients according to malaria severity. Circulating levels of IL-8 (A) and IP-10 (B) in adult malaria patients with increasing severity. HC = Healthy controls; n = 52, UCM =  uncomplicated malaria n = 45, SM =  severe malaria n = 84. Missing data one patient in each group of patients. Levels of mediators are presented as boxplots; the box shows the interquartile range, the line through the box is the median, and the whiskers indicate the 10^th^ and the 90^th^ percentiles. *p = 0.04, **p = 0.002, ***p<0.001, ****p = 0.001 (Mann-Whitney test).

Categorising the study population into healthy controls and patients with uncomplicated and severe malaria, there was a steady increase with increasing severity in those cytokines that were associated with severity, i.e., IL-8 and IP-10 (Fig. 2).

### Cytokine levels related to in-hospital mortality

During hospitalisation, ten of the malaria patients died, of which nine had a HIV co-infection. The malaria patients who died had significantly raised levels of IL-6 (p = 0.006), IL-8 (p = 0.001), MCP-1 (p = 0.012), and MIP-1β (p = 0.010) compared with the survivors.

## Discussion

In the present study, marked changes in the cytokine response during infection with *P. falciparum* were observed in malaria patients compared with healthy controls. Some of these changes, particularly the IL-8 and IP-10 levels, were strongly associated with falciparum malaria and disease severity. Both chemokines were markedly increased in patients with falciparum malaria as compared with healthy controls, and raised levels of IL-8 and IP-10 were together with low MCP-1 levels, associated with increased disease severity, even after adjusting for relevant confounders. For IL-8, particularly high levels were also found in malaria patients that were co-infected with HIV and in those who died during hospitalization.

Previous studies on the impact of cytokines on clinical outcome in malaria have identified TNF, IFN-γ, IL-1β, IL-6, IL-10, IL-12, transforming growth factor β and IP-10 as important mediators, but data on IL-8in this setting are more limited [20]–[24]. A Zambian study of falciparum malaria patients found no increased levels of IL-8, but the sample size was small, and few patients had severe malaria (8/30) [25]. In the present study, there was a strong and independent association of IL-8 with disease severity in adult falciparum malaria,. In support of our findings, in an older study from Thailand, IL-8 levels on admission were correlated with malaria parasitemia and disease severity, but the highest IL-8 levels were observed 14 days after admission, a time when viable parasites were no longer found [26]. More recently, an increased frequency of the genotype IL-8-251T/A, which leads to high IL-8 expression, has been associated with susceptibility to severe falciparum malaria in a meso-endemic area in India [27]. In children with malaria, high plasma IL-8 and IP-10 has been associated with increased severity and high cerebrospinal fluid IL-8 and IP-10 with mortality [28]–[30]. Nonetheless, clinical presentation, immune response, and mortality are often quite different in children compared with adults [31].

Several cells with relevance to severe falciparum malaria, including tissue macrophages and endothelial cells, can release IL-8 when activated. It is tempting to hypothesize that hemozoin and other parasite-related molecules can induce IL-8 release in these cells. [32]. Moreover, *P. falciparum* secretes a functional histamine-releasing factor homolog that induces IL-8 release from eosinophils [33]. The role of IL-8 with relevance for falciparum malaria include its ability to promote production of reactive oxygen species in monocytes and macrophages, to induce endothelial cell activation and to promote T cell chemotaxis and CD8^+^ T cell cytotoxicity [34]. IL-8 is a potent activator and chemo-attractant of neutrophils, and based on studies in murine models, it has also been suggested that neutrophils could be involved in sequestration and inflammation during severe malaria [35]. Moreover, a study of Nigerian children found that neutrophil extracellular traps (NETs) may induce pathology when they are infected with falciparum-malaria, but the same mechanism could potentially also be beneficial in adults by “trapping” the malaria parasites [36]. At the present, the role of neutrophils in the pathogenesis of clinical malaria is unclear. [35]. The function of the IL-8 receptor on erythrocytes has remained unclear to date, we can not exclude a role for IL-8 in erythrocyte sequestering during falciparum malaria. [37]–[39]. However, in spite of the hypothesis above, the role of IL-8 in malaria pathogenesis and protection remains unclear.

Increased levels of IP-10 have been associated with increased severity and mortality with cerebral malaria and with high viral load in HIV-infection [20], [40]. In the present study, we confirmed these findings by showing a strong and independent association between plasma levels of IP-10 and disease severity in patients with falciparum malaria. Also, in the Pelora analyses high IP-10 showed the strongest association with falciparum malaria as compared with plasma levels of healthy controls. In addition to high plasma levels of IL-8 and IP-10, low plasma levels of MCP-1 were significant associated with disease severity during falciparum malaria. We have no explanation for this finding, but interestingly, MCP-1 was suggested to be beneficial in relation to falciparum malaria in Fulani children in Mali [29], [41], and the role of MCP-1 in malaria is still unclear.

Besides IL-8, MCP-1 and IP-10, several of the cytokines that were associated with disease severity during falciparum infection belong to the CC or CXC chemokine family, i.e., eotaxin, MIP-1α, and MIP-1β. All of these are chemo-attractants for different types of leukocytes, including some with important roles in malaria pathogenesis, as the eosinophil granulocytes. Thus, increased levels of those chemokines have been associated with organ failure, such as in malaria-associated acute respiratory distress syndrome (ARDS) and severe malaria, and occasionally with mortality [20], [29], [32], [40], [42], [43].

We have previously shown that co-infection with HIV is significantly associated with increased malaria severity and death [13]. Patients co-infected with falciparum malaria and HIV had higher levels of IL-8, eotaxin and MIP-1α and lower levels of TNF compared with the patients without HIV. Eotaxin has previously been associated with anaemia in HIV-infected children with falciparum malaria [43]. IL-8 has been implicated in inflammatory responses during HIV infection as well as malaria [44]. Notably, although TNF has an established role in the pathogenesis of HIV infection and malaria, we found low rather than high levels of TNF, particularly in co-infected individuals. We have no clear explanation for this finding but it could potentially reflect that other markers such as soluble TNF receptors could be more reliable markers of TNF activity than TNF itself. It could potentially also reflect that when measuring a wide range of cytokines, trying to reflect the cytokine network, other conclusions may be obtained than when analysing one or a few cytokines. Nonetheless, this finding may underscore the complexity of the cytokine response during falciparum malaria.

The present study has some limitations. First, although most patients were severely ill, relatively few died, which limits conclusions concerning cytokine levels and mortality. Second, even if the WHO HIV clinical staging is considered an acceptable disease severity marker, the lack of information on CD4 and CD8 T cell counts is still an important limitation, in relation to the influence of HIV co-infection on the cytokine response during falciparum malaria. Third, since malaria severity tends to increase in older age groups, at least in non-endemic areas [45], the uneven age distribution may have “exaggerated” the differences in cytokine response between the patients and the healthy controls. Ideally, we should have had a selection process of the healthy controls achieving similar age groups. Fourth, assuming that tests usually done only on clinical suspicion of specific organ involvement were normal if not done, is not optimal, but still a reality in a low resource setting. Fifth, a large number of statistical tests were performed without adjusting for multiple comparisons. However, we consider it less probable that the main findings could be due purely to statistical coincidences because there was such a “systematic”, significant trend in the IL-8 and IP-10 observations. In addition, the use of a statistical method (Pelora), which is particularly suited for the actual cytokine results, limits the influence of multiple comparisons.

## Conclusions

In the present prospectively designed study with consecutive inclusion of falciparum malaria patients with and without HIV co-infection, we found marked changes in the cytokine network during malaria infection. In particular, high plasma levels of IP-10 and IL-8 were associated with falciparum malaria and disease severity in infected individuals, and for IL-8, particularly high levels were found in those that were co-infected with HIV. While several previous studies have appointed IP-10, IL-8 has been less described in relation to severe malaria.

Our findings underscore the complex role of inflammation during infection with *P. falciparum*. However, the correlations do not necessarily mean any causal relationship, and further clinical and mechanistic research is necessary to elucidate the role of cytokines in pathogenesis and protection during falciparum malaria.

## Supporting Information

S1 Figure **The correlation between the different cytokines in all malaria patients.** Pairwise Spearman correlation is given between the cytokines in all malaria patients. The darker blue, the higher correlation as seen by the correlation scale at the right. The cytokines are sorted according to groupings of highly correlated cytokines, determined by the angular order of the eigenvector.
(TIFF)

S1 File **Validation of the statistical methods presented in the manuscript.**
(DOCX)

## References

[1] Murray CJ, Rosenfeld LC, Lim SS, Andrews KG, Foreman KJ, et al (2012) Global malaria mortality between 1980 and 2010: a systematic analysis. Lancet 379:413–431. 22305225 10.1016/S0140-6736(12)60034-8

[2] Lozano R, Naghavi M, Foreman K, Lim S, Shibuya K, et al (2012) Global and regional mortality from 235 causes of death for 20 age groups in 1990 and 2010: a systematic analysis for the Global Burden of Disease Study 2010. Lancet 380:2095–2128. 23245604 10.1016/S0140-6736(12)61728-0 PMC10790329

[3] Chalwe V, van Geertruyden JP (2012) HIV-malaria co-infection: effects of malaria on the prevalence of HIV in East sub-Saharan Africa. International Journal of Epidemiology 41:: 890–891; author reply 891–892. 10.1093/ije/dys079 22617688

[4] Cohen C, Karstaedt A, Frean J, Thomas J, Govender N, et al (2005) Increased prevalence of severe malaria in HIV-infected adults in South Africa. Clinical Infectious Diseases 41:1631–1637. 16267737 10.1086/498023

[5] Berg A, Patel S, Langeland N, Blomberg B (2008) Falciparum malaria and HIV-1 in hospitalized adults in Maputo, Mozambique: does HIV-infection obscure the malaria diagnosis? Malar J 7:252. 19077302 10.1186/1475-2875-7-252 PMC2615446

[6] Iriemenam NC, Okafor CM, Balogun HA, Ayede I, Omosun Y, et al (2009) Cytokine profiles and antibody responses to Plasmodium falciparum malaria infection in individuals living in Ibadan, southwest Nigeria. Afr Health Sci 9:66–74. 19652739 PMC2707050

[7] Sun T, Holowka T, Song Y, Zierow S, Leng L, et al (2012) A Plasmodium-encoded cytokine suppresses T-cell immunity during malaria. Proc Natl Acad Sci U S A 109:E2117–2126. 22778413 10.1073/pnas.1206573109 PMC3411961

[8] Appay V, Sauce D (2008) Immune activation and inflammation in HIV-1 infection: causes and consequences. J Pathol 214:231–241. 18161758 10.1002/path.2276

[9] Ferrando-Martinez S, Ruiz-Mateos E, Romero-Sanchez MC, Munoz-Fernandez MA, Viciana P, et al (2011) HIV infection-related premature immunosenescence: high rates of immune exhaustion after short time of infection. Curr HIV Res 9:289–294. 21916840 10.2174/157016211797636008

[10] Nakanjako D, Ssewanyana I, Mayanja-Kizza H, Kiragga A, Colebunders R, et al (2011) High T-cell immune activation and immune exhaustion among individuals with suboptimal CD4 recovery after 4 years of antiretroviral therapy in an African cohort. BMC Infect Dis 11:43. 21299909 10.1186/1471-2334-11-43 PMC3065409

[11] Brentlinger PE, Behrens CB, Kublin JG (2007) Challenges in the prevention, diagnosis, and treatment of malaria in human immunodeficiency virus infected adults in sub-Saharan Africa. Archives of Internal Medicine 167:1827–1836. 17893303 10.1001/archinte.167.17.1827

[12] Macedo de Oliveira A, Mutemba R, Morgan J, Streat E, Roberts J, et al (2011) Prevalence of malaria among patients attending public health facilities in Maputo City, Mozambique. American Journal of Tropical Medicine and Hygiene 85:1002–1007. 22144434 10.4269/ajtmh.2011.11-0365 PMC3225142

[13] Berg A, Patel S, Aukrust P, David C, Gonca M, et al (2014) Increased Severity and Mortality in Adults Co-Infected with Malaria and HIV in Maputo, Mozambique: A Prospective Cross-Sectional Study. PLoS One 9:e88257. 24505451 10.1371/journal.pone.0088257 PMC3914956

[14] WHO (2011) Guidelines for the treatment of malaria, second edition.

[15] WHO (2007) HIV/AIDS Programme.

[16] Tiago AD NC, Caupers P, Mabunda S (2011) Normas de Tratamento da Malaria em Moçambique; Ministerio da Saúde DNdSP, Programa Nacional de Controlo de Malária, editor. 16 p.

[17] Genser B, Cooper PJ, Yazdanbakhsh M, Barreto ML, Rodrigues LC (2007) A guide to modern statistical analysis of immunological data. BMC Immunol 8:27. 17963513 10.1186/1471-2172-8-27 PMC2234437

[18] Dettling M BP (2004) Finding predictive gene groups from microarray data. Journal of Multivariate Analysis 90:106–131.

[19] David Hosmer SL (2000) Applied Logistic Regression; edition, editor. Danvers, MA, USA: Wiley.

[20] Jain V, Armah HB, Tongren JE, Ned RM, Wilson NO, et al (2008) Plasma IP-10, apoptotic and angiogenic factors associated with fatal cerebral malaria in India. Malar J 7:83. 18489763 10.1186/1475-2875-7-83 PMC2405803

[21] Sinha S, Qidwai T, Kanchan K, Jha GN, Anand P, et al (2010) Distinct cytokine profiles define clinical immune response to falciparum malaria in regions of high or low disease transmission. Eur Cytokine Netw 21:232–240. 21075740 10.1684/ecn.2010.0208

[22] Day NP, Hien TT, Schollaardt T, Loc PP, Chuong LV, et al (1999) The prognostic and pathophysiologic role of pro- and antiinflammatory cytokines in severe malaria. J Infect Dis 180:1288–1297. 10479160 10.1086/315016

[23] Prakash D, Fesel C, Jain R, Cazenave PA, Mishra GC, et al (2006) Clusters of cytokines determine malaria severity in Plasmodium falciparum-infected patients from endemic areas of Central India. J Infect Dis 194:198–207. 16779726 10.1086/504720

[24] Lourembam SD, Sawian CE, Baruah S (2013) Dysregulation of cytokines expression in complicated falciparum malaria with increased TGF-beta and IFN-gamma and decreased IL-2 and IL-12. Cytokine 64:503–508. 24012048 10.1016/j.cyto.2013.08.007

[25] Gamra MM, el-Sharkawy EM, Shinondo C (2001) Serum levels of some cytokines and soluble adhesion molecules in normal and patients with malignant malaria in Zambia. J Egypt Soc Parasitol 31:905–914. 11775116

[26] Burgmann H, Hollenstein U, Wenisch C, Thalhammer F, Looareesuwan S, et al (1995) Serum concentrations of MIP-1 alpha and interleukin-8 in patients suffering from acute Plasmodium falciparum malaria. Clin Immunol Immunopathol 76:32–36. 7606866 10.1006/clin.1995.1084

[27] Mahanta A, Kakati S, Baruah S (2014) The association of IL-8-251T/A polymorphism with complicated malaria in Karbi Anglong district of Assam. Cytokine 65:210–216. 24290435 10.1016/j.cyto.2013.11.001

[28] Armah HB, Wilson NO, Sarfo BY, Powell MD, Bond VC, et al (2007) Cerebrospinal fluid and serum biomarkers of cerebral malaria mortality in Ghanaian children. Malar J 6:147. 17997848 10.1186/1475-2875-6-147 PMC2186349

[29] John CC, Park GS, Sam-Agudu N, Opoka RO, Boivin MJ (2008) Elevated serum levels of IL-1ra in children with Plasmodium falciparum malaria are associated with increased severity of disease. Cytokine 41:204–208. 18282763 10.1016/j.cyto.2007.12.008 PMC2323512

[30] Erdman LK, Dhabangi A, Musoke C, Conroy AL, Hawkes M, et al (2011) Combinations of host biomarkers predict mortality among Ugandan children with severe malaria: a retrospective case-control study. PLoS One 6:e17440. 21364762 10.1371/journal.pone.0017440 PMC3045453

[31] Hawkes M, Elphinstone RE, Conroy AL, Kain KC (2013) Contrasting pediatric and adult cerebral malaria: the role of the endothelial barrier. Virulence 4:543–555. 23924893 10.4161/viru.25949 PMC5359751

[32] Gillrie MR, Lee K, Gowda DC, Davis SP, Monestier M, et al (2012) Plasmodium falciparum histones induce endothelial proinflammatory response and barrier dysfunction. Am J Pathol 180:1028–1039. 22260922 10.1016/j.ajpath.2011.11.037 PMC3448071

[33] MacDonald SM, Bhisutthibhan J, Shapiro TA, Rogerson SJ, Taylor TE, et al (2001) Immune mimicry in malaria: Plasmodium falciparum secretes a functional histamine-releasing factor homolog in vitro and in vivo. Proc Natl Acad Sci U S A 98:10829–10832. 11535839 10.1073/pnas.201191498 PMC58559

[34] Clark IA, Schofield L (2000) Pathogenesis of malaria. Parasitol Today 16:451–454. 11006479 10.1016/s0169-4758(00)01757-9

[35] Ioannidis LJ, Nie CQ, Hansen DS (2014) The role of chemokines in severe malaria: more than meets the eye. Parasitology 141:602–613. 24476686 10.1017/S0031182013001984 PMC3962270

[36] Baker VS, Imade GE, Molta NB, Tawde P, Pam SD, et al (2008) Cytokine-associated neutrophil extracellular traps and antinuclear antibodies in Plasmodium falciparum infected children under six years of age. Malar J 7:41. 18312656 10.1186/1475-2875-7-41 PMC2275287

[37] Patnaik JK, Das BS, Mishra SK, Mohanty S, Satpathy SK, et al (1994) Vascular clogging, mononuclear cell margination, and enhanced vascular permeability in the pathogenesis of human cerebral malaria. Am J Trop Med Hyg 51:642–647. 7985757

[38] McMorran BJ, Marshall VM, de Graaf C, Drysdale KE, Shabbar M, et al (2009) Platelets kill intraerythrocytic malarial parasites and mediate survival to infection. Science 323:797–800. 19197068 10.1126/science.1166296

[39] Horuk R (1994) The interleukin-8-receptor family: from chemokines to malaria. Immunol Today 15:169–174. 8198708 10.1016/0167-5699(94)90314-X

[40] Ochiel DO, Awandare GA, Keller CC, Hittner JB, Kremsner PG, et al (2005) Differential regulation of beta-chemokines in children with Plasmodium falciparum malaria. Infect Immun 73:4190–4197. 15972509 10.1128/IAI.73.7.4190-4197.2005 PMC1168587

[41] Bostrom S, Giusti P, Arama C, Persson JO, Dara V, et al (2012) Changes in the levels of cytokines, chemokines and malaria-specific antibodies in response to Plasmodium falciparum infection in children living in sympatry in Mali. Malar J 11:109. 22480186 10.1186/1475-2875-11-109 PMC3366880

[42] Mohan A, Sharma SK, Bollineni S (2008) Acute lung injury and acute respiratory distress syndrome in malaria. J Vector Borne Dis 45:179–193. 18807374

[43] Davenport GC, Hittner JB, Were T, Ong'echa JM, Perkins DJ (2012) Relationship between inflammatory mediator patterns and anemia in HIV-1 positive and exposed children with Plasmodium falciparum malaria. Am J Hematol 87:652–658. 22570198 10.1002/ajh.23200 PMC4703404

[44] Ronsholt FF, Ullum H, Katzenstein TL, Gerstoft J, Ostrowski SR (2013) Persistent inflammation and endothelial activation in HIV-1 infected patients after 12 years of antiretroviral therapy. PLoS One 8:e65182. 23755191 10.1371/journal.pone.0065182 PMC3670840

[45] Muhlberger N, Jelinek T, Behrens RH, Gjorup I, Coulaud JP, et al (2003) Age as a risk factor for severe manifestations and fatal outcome of falciparum malaria in European patients: observations from TropNetEurop and SIMPID Surveillance Data. Clinical Infectious Diseases 36:990–995. 12684911 10.1086/374224

