# Supplementary figures and images for: Cytokine Network in Adults with Falciparum Malaria and HIV-1: Increased IL-8 and IP-10 Levels Are Associated with Disease Severity
Source: PLoS One. 2014 Dec 11;9(12):e114480. doi: 10.1371/journal.pone.0114480 (PMC4263737; doi:10.1371/journal.pone.0114480)

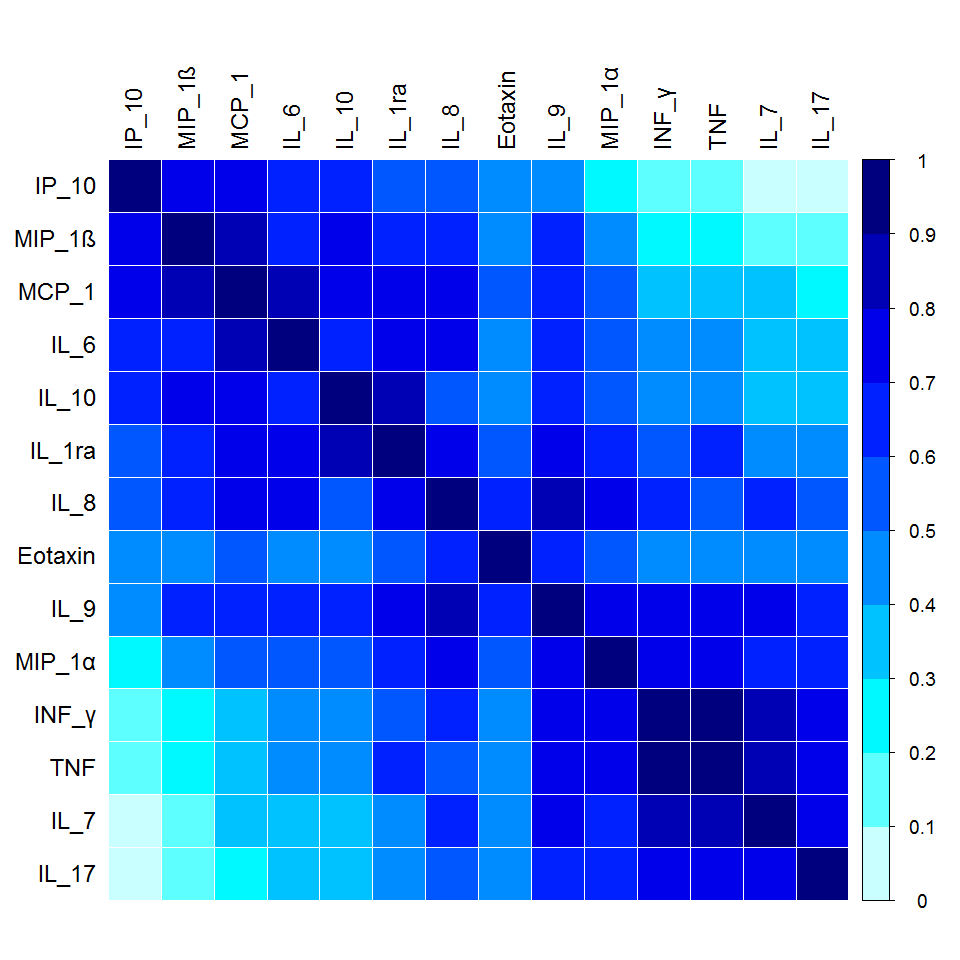

Supplement: S1 Figure — The correlation between the different cytokines in all malaria patients. Pairwise Spearman correlation is given between the cytokines in all malaria patients. The darker blue, the higher correlation as seen by the correlation scale at the right. The cytokines are sorted according to groupings of highly correlated cytokines, determined by the angular order of the eigenvector. (TIFF) [file pone.0114480.s001.tiff]
