# Supplementary material for: Cytokine Network in Adults with Falciparum Malaria and HIV-1: Increased IL-8 and IP-10 Levels Are Associated with Disease Severity
Source: PLoS One. 2014 Dec 11;9(12):e114480. doi: 10.1371/journal.pone.0114480 (PMC4263737; doi:10.1371/journal.pone.0114480)
Supplement: S1 File — Validation of the statistical methods presented in the manuscript. (DOCX) [file pone.0114480.s002.docx]

**File S1:**

**Validation of the statistical methods presented in the manuscript:**

Most of the methods used in the article are robust non-parametric methods which require few assumptions.

**1. The Chi-square tests for independence** was used to test for differences in categorical variables such as sex and HIV-status. A requirement for this test is that at least 80% of the cells have an expected count of at least 5, and there were no cells with an expected count of less than five in the situations where we applied this test.

**2. The Mann Whitney test** was used to test for differences in continuous variables between two independent groups. This test requires independent measurements in each group, i.e. independence between the subjects in each group, which is fulfilled as we have a random sample of patients and controls among those meeting the inclusion criteria. We needed to use the Mann Whitney test as many of the variables, in particular the cytokine measurements, have a highly non-normal distribution. I.e., t-tests would not be appropriate.

3. **Spearman rank correlation** is used for calculating correlations between cytokines, due to the very skew distribution of the cytokine values and some outliers. Spearman rank correlation is robust against outliers and skew distributions by being based on the rank of the data.

In addition to univariable analyses with the Chi-square and the Mann-Whitney test, we wanted to look into which combinations of variables could best distinguish between patient groups (and between patients and controls). In particular we wanted to consider which combinations of cytokines that could describe differences between patient groups (and patients versus controls). A challenge for such multivariable analyses is that cytokines are highly correlated. Supplementary 1, Figure A1 shows the correlations among the various cytokines for all malaria patients. Moreover, we also have a relatively high number of cytokines to consider compared to the number of patients. These challenges, common in cytokine data [17], imply that for instance ordinary multivariable logistic regression that otherwise would be a common method to use will run into problems here.

4. **The penalized logistic regression analysis**, **Pelora** appoints groups of cytokines characterizing just the property we are searching for, as which group of cytokines are characteristic for all malaria patients compared to healthy controls, for HIV positive malaria patients compared to the HIV negative malaria patients and for those with severe malaria compared to the patients with uncomplicated malaria [18]. The strength of the influence of the given cytokine on the group property is given as the “predictive ability”, where the closer to the number 1, the better. The method is particularly suited for data with high correlation and many observations in relatively few individuals. Said in more professional statistical terms, Pelora is a method constructed for supervised clustering in situations with strong correlations among predictors, and many predictors compared to the number of observations. I.e., this method is constructed exactly for analyses of data with those challenges (many highly correlated predictors) we have in our data. The method finds clusters of predictors which are good at predicting which group each measurement belong to. In our case meaning groups of cytokines which are good at predicting which group each patient belongs to. The method proceeds in a stepwise forward manner by first identifying a first cluster of cytokines that predict as much as possible, then a second cluster, which corrects as many as possible of the remaining misclassifications, and so on until a certain stopping criteria. The usual stopping criteria is to stop when the decrease in prediction error flattens out [2]. Typically, at least in our applications, the first cluster explains a lot of the difference between the groups, and looking at which cytokines are selected in this group is thus of particular interest.

Pelora is also considered to be a quite robust method requiring few assumptions on the data. One assumption for the method to work reasonably is that the distribution of the predictor variables, after they are standardized by subtracting the mean and dividing by the standard deviation are relatively similar and without extreme outliers. To achieve this in our case we first log transform the data and reset a few (1-2 for a few of the cytokines) extreme outliers to three standard deviations above the mean. Then we checked with boxplots of the standardized log-transformed cytokine values for all malaria patients and for all controls. We see that they have a fairly similar distribution and there are no extreme outliers.


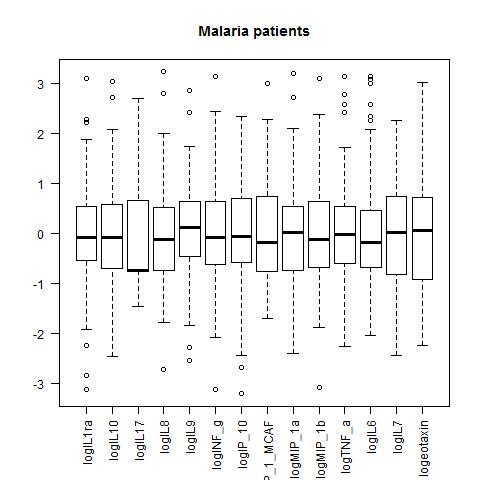


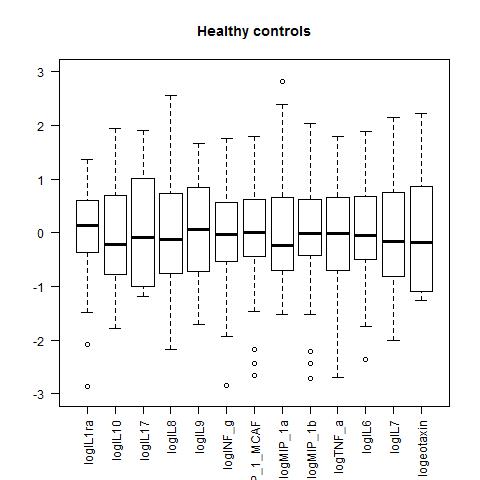


Further there is a tuning parameter λ in the Pelora method which needs to be selected. This parameter determines the amount of penalization in the penalized likelihood criterion which Pelora optimizes, see [2]. We have run all analyses with the default setting λ=1/32 [2], but to validate the impact of this choice we have rerun all analyses with a sequence of different λ values. Looking at which cytokines are selected in the first cluster, which is of particular interest to us, we found that the same selection is made over a broad range of λ-values. In the analyses comparing different groups of malaria patients any λ from 0 to more than 10 give the same cytokine selections. In the analyses, comparing malaria patients with controls the selection is stable for λ values from 1/64 to more than 10, while half of the cytokines are not selected if a λ less than 1/64 is used. I.e., the results are very robust to the choice of λ.

Due to the limited number of patients, and since we were interested studying how cytokines combined in clusters explaining the difference between groups rather than predicting group membership of future patients, we fitted Pelora on all data. The reported predictive ability (measured as area under the ROC curve, AUC) is thus based on predicting the outcome on the training data, usually resulting in somewhat too optimistic values. To examine this we finally looked at the obtained predictive ability when we split the data in two parts. We used a random 2/3 of the data to fit the model and the remaining 1/3 to calculate the predictive ability. We repeated this 1000 times and calculated the average predictive ability. For the case with malaria patients versus controls the obtained AUC were only negligibly smaller than with the training data, less than 1% lower. For the two cases with two different groups of malaria patients the obtained AUC were 10-15% lower than on the training data. Taken into account that we expect the predictive ability to be a bit better when the model is fitted to the entire dataset instead of just 2/3 the reported predictive abilities does not seem to be notably too optimistic for what one would see on future data.

Output files from SPSS and R can be obtained from the first author.
